# Developing Catalysts for the Hydrolysis of Glycosidic Bonds in Oligosaccharides Using a Spectrophotometric Screening Assay

**DOI:** 10.1021/acscatal.4c03261

**Published:** 2024-08-14

**Authors:** Susanne Striegler

**Affiliations:** Department of Chemistry and Biochemistry, University of Arkansas, Fayetteville, Arkansas 72701, United States

**Keywords:** nanogels, glycoside, carbohydrate, oligosaccharide, hydrolysis, catalysis, screening assay

## Abstract

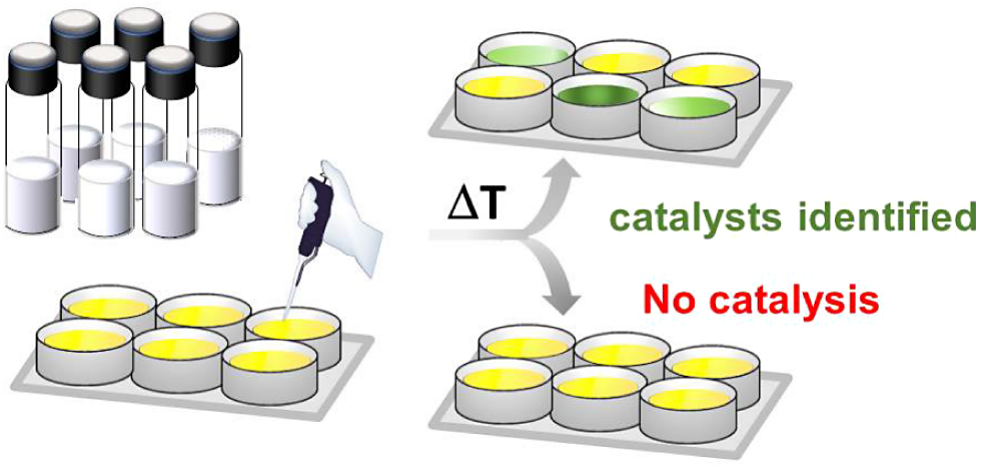

In a proof-of-concept study, a method for the empirical design of polyacrylate gel catalysts with the ability to cleave 1→4 α-glycosidic bonds in di- and trisaccharides was elaborated. The study included the synthesis of a 300-gel member library based on two different cross-linkers and 10 acrylate monomers, identification of monomodal gels by dynamic light scattering, and a 96-well plate spectrophotometric screening assay to monitor the hydrolysis of chromophore-free maltose into glucose units. The composition of the matrix of the most efficient catalysts in the library was found to enable CH−π, hydrophobic, and H-bond accepting interactions during the hydrolysis as typically seen in glycosylases. The same gel catalysts allowed the hydrolysis of the trisaccharide maltotriose with a catalytic proficiency of 2 × 10^6^ indicating transition state stabilization during the hydrolysis of 5 × 10^–7^. The results place the developed gels among the most efficient catalysts developed for the hydrolysis of natural saccharides. The elaborated strategy may lead to catalysts that can transform polysaccharides into valuable synthons in the near future.

## Introduction

Starch hydrolysis plays a vital role in diverse industries contributing to the production of a wide range of products, including food ingredients,^1,2^ sustainable packing materials,^3^ biofuels,^4^ chemicals, and pesticide formulations,^5^ carriers of pharmaceuticals,^6^ and during the sustainable valorization of textile wastes.^7^ An efficient breakdown of its complex structure into simpler carbohydrates, primarily the monosaccharide glucose, enables the utilization of starch as a renewable and versatile raw material. Methods to accomplish this goal include enzymatic hydrolysis with amylases, glucoamylases, and pullulanases^8^ and/or hydrolysis with strong acids in combination with heat treatment,^1,5^ microbial fermentation,^2^ and ultrasonic treatment.^4^ While enzymatic starch hydrolysis is particularly appealing due to its ease of use and advantages, including mild reaction conditions and high efficiency, the use of enzymes comes with some disadvantages. These shortcomings can, among others, encompass their costs for use in large quantities, temperature and pH sensitivity, long reaction times, possible inactivation, denaturation, product inhibition, microbial contamination, and the inherent substrate specificity that may require the use of an enzyme cocktail to complete the hydrolysis reaction.^2,8^

An alternative to the use of enzymes may be found in biomimetic and enzyme-like catalysts targeting the transformation of glycosidic bonds.^9−14^ In this context, catalysts that combine elements of transition metal catalysis with the effects of matrix-supported secondary interactions thereby stabilizing the transition state of the targeted reaction are of particular interest.^2,15,16^ Rationally developing man-made catalysts for the hydrolysis of nonactivated carbohydrates requires detailed knowledge of the reaction mechanism, iterative adjustment of the catalyst design and composition, various syntheses and physical characterizations, testing of catalytic performance, and potentially scale-up and commercialization. When opting for empirical catalyst design, screening for selected properties, parameter optimization, and an iterative process of trial-and-error experimentation are often corner stones of research efforts. In the latter scenario, the initial catalyst choice relies on a structure with a known efficiency toward the targeted reaction.

In this context, we established micro- and nanogels with immobilized metal complexes as a platform for the hydrolysis of glycosidic bonds over the past years.^11,12,17−22^ Early efforts focused on a rational development of these catalysts by determining the contributions of the metal complex core and the surrounding matrix based on the synthesis conditions in ionic and nonionic surfactants.^18−22^ Those early attempts focused on the hydrolysis of activated glycosidic bonds in glycosides with chromophores to allow spectrophotometric and fluorometric analyses.^18−22^ By contrast, recent work targets the hydrolysis of glycosidic bonds in nonactivated disaccharides aiming at the design of catalysts that eventually may be used for the hydrolysis of oligo- and polysaccharides.^11,12,17^

**Chart 1:**
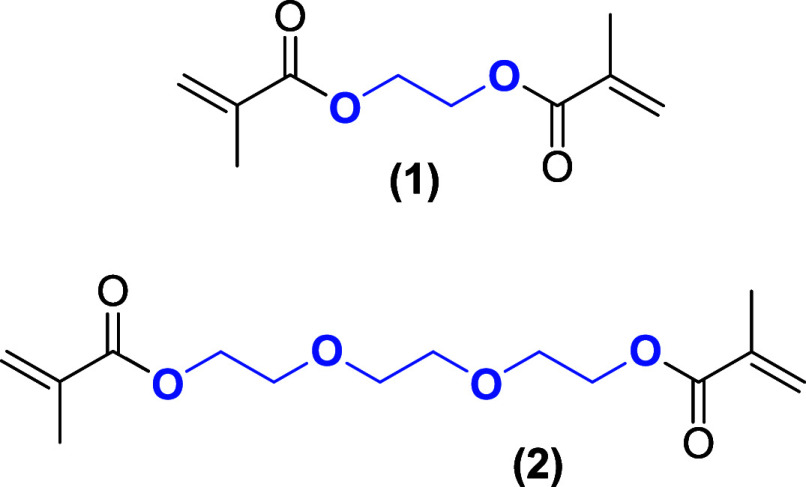
Structures of the Cross-Linkers EGDMA (**1**) and TEGDMA (**2**)

Though kinetic data could not be obtained, nanogel-catalyzed hydrolyses of glycosidic bonds in common disaccharides were recently achieved.^12^ In more detail, up to 18 μg L^–1^ glucose is formed from maltose in hydrolysis assays that heat the gel–sugar mixture to 60 °C over 72 h.^12^ In parallel efforts, a 12-well plate assay for rapid gel synthesis was developed and coupled with dynamic light scattering analysis to determine the cross-linking content of gels leading to efficient disaccharide hydrolysis.^11^ The study showed comparable performance of polymers made from EGDMA (**1**, 60 mol %) and TEGDMA (**2**, 25 mol %) using butyl acrylate as a comonomer for the hydrolysis of turanose. The different length of the ethylene glycol bridge between the acrylate units in the cross-linkers is the foundation for a higher probability for TEGDMA (**2**) than for EGDMA (**1**) to form H-bond accepting interactions (Chart 1). Branched or less polar di-, tri-, penta-, and hexa(meth)acrylates used in the same study yielded less efficient catalysts.^11^ In order to develop catalysts with high efficiency to cleave a 1→4 α-glycosidic bond, a library of 300 gels with unique composition is developed here using empirical catalyst design. The study identifies biomimetic catalysts for the hydrolysis of nonactivated glycosidic bonds in natural saccharides and may thereby open a new path toward transforming and utilizing biomass.

## Results and Discussion

### Nanogel Synthesis

**Chart 2:**
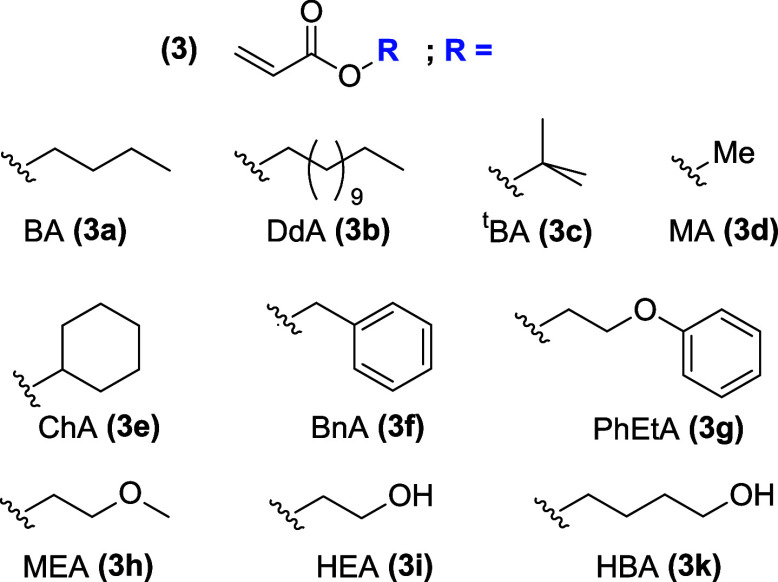
Structures of Acrylate Monomers (**3**)

Taking advantage of the previous results,^11^ two series of gels using cross-linkers **1** and **2** are synthesized in 12-well plates generating a library of 300 polyacrylate gels with unique composition. While the amounts of cross-linkers are kept constant at previously determined molar amounts,^11^ 40 mol % of polymerizable acrylate monomers (**3**) are altered for the synthesis of EGDMA-containing gels and 75 mol % for TEGDMA-containing gels. The nature, amount, and composition of 10 acrylate monomers are systematically altered during gel synthesis (Chart 2). Structural differences in the acrylate monomers (**3**) are the foundation for their ability to provide secondary interactions with potential substrates including hydrophobic interactions, π–π stacking, CH−π stacking, and hydrogen bond-donating and -accepting interactions.^19^ A potpourri of these interactions is typically found in the active sites of glycosylases and inspired the choices of monomers employed here. While theoretically infinite, the systematic variation of **3** finds a practical lower limit in the volumes that can be reliably measured. The smallest amount of monomer in prepolymerization mixtures is arbitrarily set to 10 mol % for EGDMA-containing gels and to 12.5 mol % for those containing TEGDMA (see Supporting Information). To obtain proof of principle in this study, all gels contain up to four different monomers **3** when using EGDMA, and at most, three different monomers when synthesized from TEGDMA.

**Chart 3:**
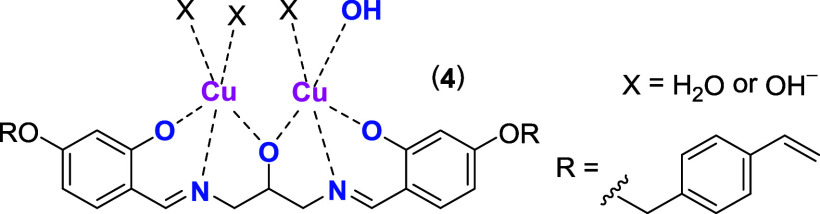
Structure of the Catalytic Metal Complex Core in an Aqueous Alkaline Buffer at pH 10.5

**Scheme 1:**
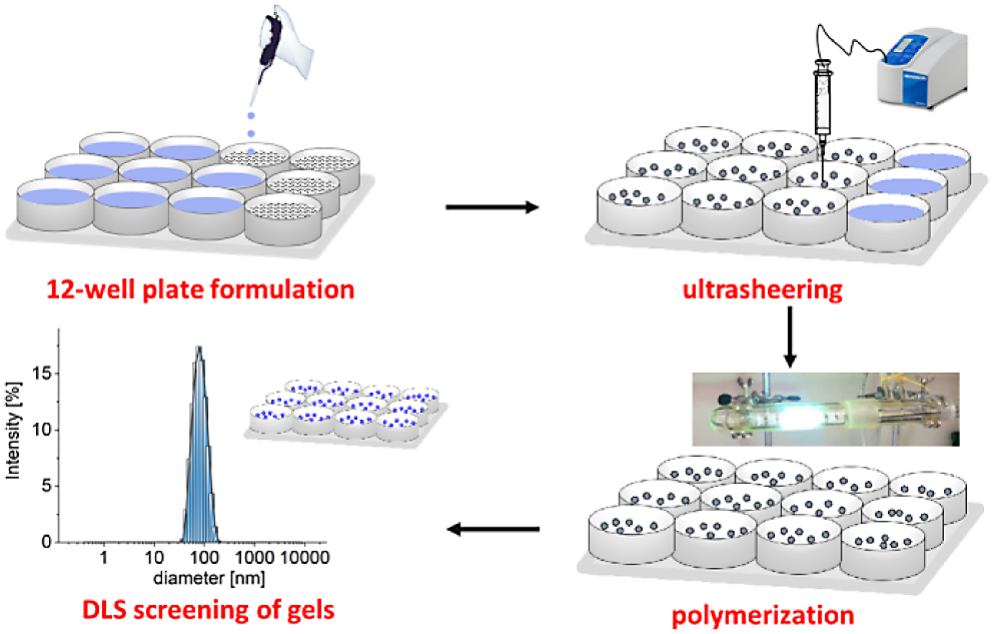
Nanogel Synthesis and Screening for Size and Homogeneity

The photoinitiated polymerization of the prepolymerization mixtures proceeds in 12-well plates as described (Scheme 1).^11^ The polyacrylate gels contain an overall 0.35 mmol polymerizable acrylate (cross-linker and monomer combined) and 0.5 mol % of Cu_2_VBbsdpo complex as a catalytic core in 2 mL TWEEN 80/SPAN 80/CAPS surfactant/buffer solution per well.^11^ Each polymer composition was synthesized in duplicate. The catalytic core of the gels is *in situ* formed during polymerization from pentadentate ligand VBbsdpo (**4**), Cu(II) ions and mannose as a counterion as described (Chart 3).^23^ The components are added to the acrylates as a premade mixture in a molar ratio of ligand **4**: Cu(II):counterion as 1:2:5 as described.^19,21,23,24^ The excess of mannose ensures near complete coordination of the sugar during material synthesis.^25^ Thereby, the potential leaching of Cu(II) ions from the metal complex is prevented. Consequently, the paramagnetic metal ions do not interfere with the radical polymerization reaction during material synthesis. As the nonionic surfactants are nondialyzable, none of the synthesized gels can be analyzed for elemental composition or substrate accessible surface areas. However, the dialysis purified all gels from unreacted monomers and sugar counterions and allowed analysis of their seize distribution and dispersity by dynamic light scattering as described (see Supporting Information).^11^ The dialysis and subsequent reloading of metal ions into the catalytic core activates the gels for catalytic hydrolysis.^18^ Typically, the sugar counterion is thereby replaced by hydroxyl ions and water molecules depending on the pH value of the solution.^23^

### Nanogel Screening for Size and Homogeneity

The synthesized gels are purified and prepared for analysis by dynamic light scattering to determine their hydrodynamic diameter and dispersity as described.^11,17,18^ In short, a 200 μL aliquot of each gel is diluted with nanopure water to 1000 μL and subjected to repetitive extraction with excess 1,2-dichloroethane. Subsequently, the aqueous layer is diluted with nanopure water to yield an overall 1250-fold diluted solution of the original gel aliquot. The *Z*-average and the dispersity of each sample are then determined at 20 °C using dynamic light scattering (DLS).

The polymer library consists of 300 gels with unique compositions: 96 gels are synthesized from cross-linker **1** and 204 from **2**. Among those, the DLS analysis identified 24 monomodal gels with EGDMA and 87 with TEGDMA backbone. The data for all monomodal gels are given as an average of 5–10 measurements (see Supporting Information). The dispersity of the selected gels is narrow or moderate with dispersity indices between 0.09 and 0.35. Monomodal gels were previously shown to have generally higher catalytic efficiency for glycoside hydrolyses than their bi- or polymodal counterparts.^17,18^ Particularly nanogels with a diameter significantly below 100 nm were found to be highly effective for the hydrolysis of glycosidic bonds.^18^

### Screening for Hydrolytic Activity of Monomodal Gels

Without prior purification, aliquots of 111 selected monomodal gels are employed for a newly developed spectrophotometric assay. Using maltose as a substrate, gel compositions for the cleavage of 1→4 α-glycosidic bonds with high catalytic efficiency are thereby identified. The colorimetric assay is based on literature accounts for the identification of aldoses using toluidine in acetic acid and 1,3-propanediol,^26−28^ and transformed here for use in 96-well plates.

The two-step assay begins with the hydrolysis of 25 μL of 50 mM maltose in mixtures with 25 μL aliquots of polyacrylate gel suspension that are diluted prior to use with nanopure water (1/4; v/v).^11,12^ The hydrolysis is conducted at 60 °C in 180 μL of nanopure water and initiated by the addition of 20 μL of 10 mM aqueous sodium hydroxide solution. After 24 h, the resulting solutions are flash-frozen and stored at −20 °C until use. In a second step, a 25 μL aliquot of the thawed sugar/gel mixture is heated to 120 °C with a 100 μL aliquot of the toluidine reagent in a 96-well plate for 20 min. After cooling to ambient temperature, the absorbance is read at 620 nm, and an image of the plate is taken to preserve the color distribution (see Supporting Information).

**Scheme 2:**
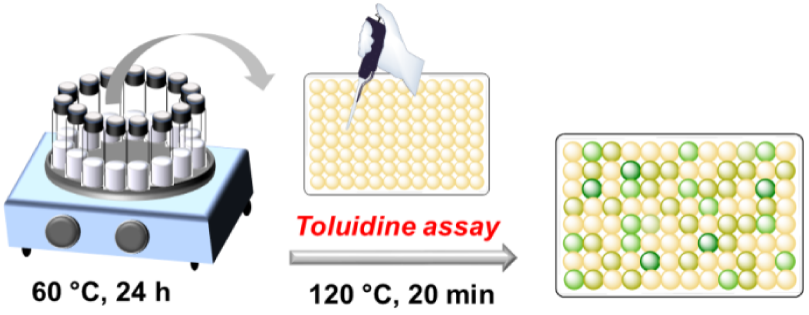
Visualization of the Hydrolytic Catalyst Activity with a Colored Screening Assay

The glucose hydrolysis product reacts with the toluidine reagent yielding a green solution of glucosyl-amine and Schiff base.^29^ The darker the green color, the more glucose was formed during the hydrolysis of maltose. While the toluidine reagent is said to be specific for aldoses, it will also react with the reducing moiety of the disaccharide starting material. However, the arbitrary units of the absorbance for the toluidine–glucose complex are, at the same concentration, 6-fold higher than for the same complex formed from maltose at 620 nm (see Supporting Information). The spectrophotometric assay serves in this study as a qualitative measure to identify gels with high efficiency for the hydrolysis of the targeted 1→4 α-glycosidic bond. On a side note, the sensitivity of the naked eye for color shades and tones is typically enough to classify the color distribution and intensity over the plate (see Supporting Information). Thus, the naked eye is adequate to determine the most efficient gels for targeted hydrolysis (Scheme 2). If the gels are ineffective catalysts for the targeted hydrolysis of maltose, a yellowish color persists after heating.

The toluidine screening assay of the maltose hydrolysis assay indicates a wide spectrum of the catalytic activity of the examined polyacrylates. Most gels show absorbance readings between 0.12 and 0.25 at 620 nm and are not further considered in the context of this study. Absorbance readings over 0.30 are observed for only 5 of the 111 gels in the library. These gels and 4 additional control polymers are then evaluated for their catalytic performance by kinetic assays (see below). Control experiments with hydrolytic gels synthesized from cross-linker **1** and butyl acrylate give absorbance reads of 0.11–0.12 au at 620 nm. Gels prepared from **2** behave likewise (Δ*A*_620 nm_ = 0.10–0.11 au). Thus, catalysts synthesized from cross-linker and butyl acrylate yield polyacrylates with a putatively lower catalytic activity than gels prepared here by systematic variation of the monomer content. Control experiments of sugar hydrolyses without catalyst give absorbance reads of 0.06 au, while the toluidine reagent by itself displays absorbance reads of 0.04 au at 620 nm after heating. All absorbance data are given as an average of at least two independent assays, not corrected for background reactions, and analyzed for qualitative information only to deduce the most potent catalysts in the library (see Supporting Information).

### Gel-Catalyzed Maltose Hydrolysis

Prior to kinetic analyses, aliquots of the selected 9 gels are purified by dialysis and thereby diluted 1 to 4 as described.^12,18^ As the elaborated 30 min polymerization assays in 12-well plates ensure near quantitative formation of gels by free radical polymerization, the concentrations of the resulting gel stock solutions are based on the theoretical amount of immobilized metal complex.^11^ Consequently, the final concentration of the gel catalysts equals 0.175 mM in all kinetic assays that are done as described.^11^

In short, 250 μL aliquots of the purified and diluted gel solutions are added to 250 μL aliquots of maltose stock solutions diluted in 1800 μL of water. The maltose concentrations in the assay are between 2.5 and 50 mM. The hydrolysis is initiated by the addition of a 200 μL aliquot of 10 mM aqueous sodium hydroxide solution at 60 °C. Over a 90 min time period, 100 μL aliquots from the reaction mixture are taken at 15 min intervals, added to centrifuge vessels prefilled with 100 μL of 12.88 mM aqueous hydrogen chloride solution to neutralize the base, flash frozen in liquid nitrogen, and stored at −20 °C until use.

**Figure 1:**
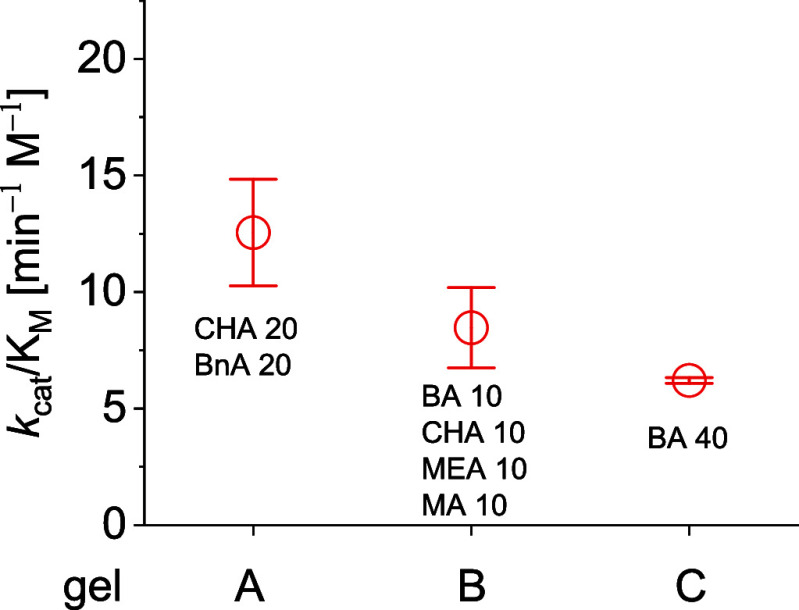
Catalytic efficiency of selected polyacrylate gels with EGDMA backbone (60 mol %) toward the hydrolysis of maltose into glucose units.

**Figure 2:**
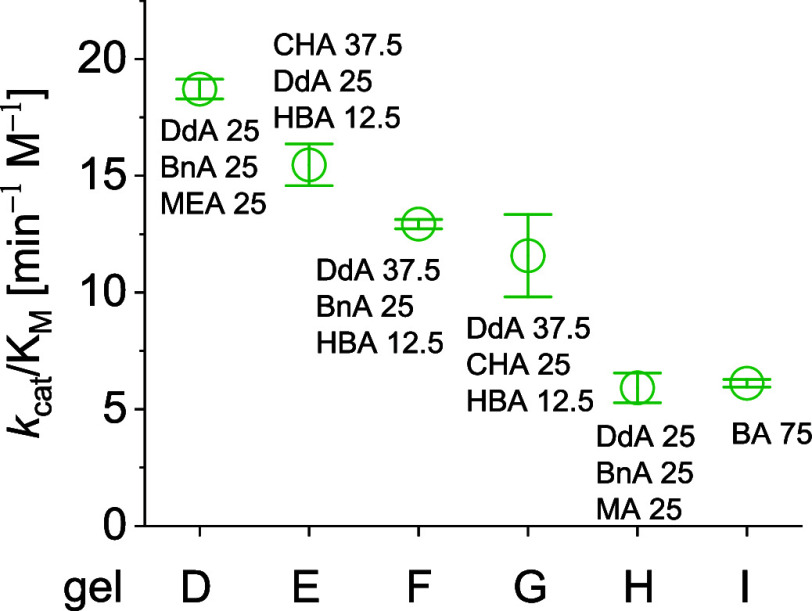
Catalytic efficiency of selected polyacrylate gels with TEGDMA backbone (25 mol %) toward the hydrolysis of maltose into glucose units.

The subsequent analysis and quantification of the sugar content of each aliquot by HPLC used an amino column as the stationary phase and 80% aqueous acetonitrile at 1 mL/min as the mobile phase.^11^ The gel-catalyzed maltose hydrolysis is monitored by the formation of glucose (*R*_t_ = 7.4 min) and quantified by integration of the peak area. Concentrations are derived by comparison to a glucose calibration curve. The time-dependent concentration is corrected for background hydrolysis and plotted over the substrate concentration. The resulting hyperbolic data are fitted by nonlinear regression to deduce the rate constant (*k*_cat_) and the Michaelis–Menten constant (*K*_M_). The maltose hydrolysis was followed likewise in the absence of polyacrylate catalysts yielding a linear correlation over the evaluated concentration range allowing us to deduce the rate constant of the uncatalyzed reaction (*k*_non_) (Table 1). Gels synthesized from EGDMA cross-linker **1** (Figure 1) are overall less efficient in the hydrolysis of 1→4 α-glycosidic bonds than gels synthesized from **2** (Figure 2). A rationale for this observation might be the found in the different amount of cross-linking content used to synthesize the gels, and in the nature of the cross-linkers themselves. Inherently, gels synthesized from 60 mol % of EGDMA can at most contain 40 mol % of variable monomer content. By contrast, the use of 25 mol % of TEGDMA allows the incorporation of 75 mol %, *i.e.*, almost twice as much, of variable monomers to enable and support secondary interactions of the gels during the catalysis. The gels synthesized from **2** thus offer a much larger opportunity to introduce a wider spectrum of secondary hydrolysis-supporting interactions than the gels synthesized from **1**.

**Table 1:** Kinetic Parameter for the Gel-Catalyzed Hydrolysis of Maltose at 60 °C^a^

gel	monomer composition (mol %)	*k*_cat_ ± Δ*k*_cat_ (min^–1^)	*K*_M_ ± Δ*K*_M_ (mM)	*k*_cat_/*K*_M_ [min^–1^ M^–1^)	*k*_cat_/(*K*_M_ × *k*_non_)
EGDMA cross-linker (60 mol %)					
A	BnA/CHA—20/20	0.058 ± 0.011	4.70 ± 0.32	12.5 ± 2.3	930,000
B	BA/CHA/MEA/MA—10/10/10/10	0.046 ± 0.009	5.50 ± 0.35	8.5 ± 1.7	630,000
C	BA—40	0.091 ± 0.002	14.7 ± 0.83	6.2 ± 0.1	460,000
TEGDMA cross-linker (25 mol %)					
D	DdA/BnA/MEA—25/25/25	0.134 ± 0.003	7.16 ± 0.41	18.7 ± 0.4	1,390,000
E	CHA/DdA/HBA—37.5/25/12.5	0.048 ± 0.003	3.10 ± 0.66	15.5 ± 0.9	1,150,000
F	DdA/BnA/HBA—37.5/25/12.5	0.024 ± 0.001	1.83 ± 0.16	12.9 ± 0.2	960,000
G	DdA/CHA/HBA—37.5/25/12.5	0.046 ± 0.007	4.00 ± 2.44	11.6 ± 1.8	860,000
H	DdA/BnA/MA— 25/25/25	0.036 ± 0.004	6.10 ± 2.10	5.9 ± 0.6	440,000
I	BA—75	0.120 ± 0.003	19.7 ± 1.6	6.1 ± 0.2	450,000

a In the presence of aqueous 0.8 mM NaOH; *k*_non_ = 1.30 × 10^–5^ min^–1^  M^–1^.

Interestingly, the most efficient gels in both series (gels A and D) contain 20–25 mol % of benzyl acrylate monomer, indicating the importance of CH−π interactions during glycoside hydrolysis. Hydrophobic interactions provided by dodecyl acrylate or cyclohexyl acrylate monomers strongly support the catalytic hydrolysis ability of the resulting gels as well (gels B and E). These findings remind us of the composition of active sites in natural glycosylases where, aside from the catalytic glutamate and aspartate residues, amino acids with aromatic and nonpolar side chains play key roles in the catalytic turnover. By contrast, acrylate monomers offering hydrogen bond-donating and -accepting interactions (gels F–H) make smaller contributions to the catalytic efficiency of the corresponding gels. However, the employed cross-linkers already support H-bond-accepting interactions and may thus overwrite the effect of such monomers.

Over all, the catalytic efficiency of the gels synthesized from mixtures of monomers is up to 2-fold higher for gels prepared from EGDMA (Table 1, gel A *versus* C) and up to 3-fold higher for gels prepared from TEGDMA compared to those previously synthesized from butyl acrylate monomer only (Table 1, gel D *versus* I). Thus, secondary interactions make major contributions during the catalytic transformation and play key roles in stabilization of the transition state. As an independent confirmation of previous results,^11^ the gels derived from different cross-linkers and butyl acrylate have comparable catalytic efficiency and proficiency for the hydrolysis of the targeted glycosidic bond.

### Gel-Catalyzed Maltotriose Hydrolysis

With efficient catalysts for the hydrolysis of 1→4 α-glycosidic bonds on hand, the cleavage of oligo- and polysaccharides containing such bonds into defined sugar units is within reach. As a first step toward this end, the gel-catalyzed hydrolysis of maltotriose is examined. The trisaccharide consists of three glucose units that are linked by 1→4 α-glycosidic bonds. For a kinetic analysis, the cleavage of one glycosidic bond was monitored by following the formation of maltose using the described hydrolysis and HPLC assays (see above). The formation of maltose (*R*_t_ = 15.2 min) is quantified by comparison to a calibration curve. The rate of maltose formation over time is then plotted over the maltotriose concentration. The resulting hyperbolic data are analyzed using the Michaelis–Menten model to deduce kinetic parameters (Table 2). The uncatalyzed reaction is monitored under similar conditions in the absence of a catalyst. When plotting the formation of maltose over time in correlation with the original maltotriose concentration, a linear correlation results from which the uncatalyzed rate constant is deduced.

**Table 2:** Kinetic Parameters for the Gel-Catalyzed Hydrolysis of Maltotriose at 60 °C^a^

gel	monomer composition (mol %)	*k*_cat_ ± Δ*k*_cat_ (min^–1^)	*K*_M_ ± Δ*K*_M_ (M)	*k*_cat_/*K*_M_ (min^–1^ M^–1^)	*k*_cat_/(*K*_M_ × *k*_non_)
EGDMA cross-linker (60 mol %)					
A	CHA/BnA—20/20	0.012 ± 0.003	0.007 ± 0.002	1.63	1,300,000
B	CHA/BA/MEA/MA—10/10/10/10	0.018 ± 0.007	0.009 ± 0.004	1.94	210,000
C	BA—40	0.0009 ± 0.0003	0.016 ± 0.008	0.06	43,000
TEGDMA cross-linker (25 mol %)					
D	DdA/BnA/MEA—25/25/25	0.012 ± 0.001	0.005 ± 0.001	2.60	2,000,000
E	CHA/DdA/HBA—37.5/25/12.5	0.020 ± 0.001	0.009 ± 0.001	2.20	1,800,000
F	DdA/BnA/HBA—37.5/25/12.5	=^b^	=^b^	=^b^	=^b^
G	DdA/CHA/HBA—37.5/25/12.5	0.048 ± 0.004	0.028 ± 0.003	1.70	1,400,000
H	DdA/BnA/MA—25/25/25	0.017 ± 0.001	0.014 ± 0.001	1.20	940,000
I	BA—75	0.027 ± 0.008	0.890 ± 0.015	0.03	24,000

a In the presence of aqueous 0.8 mM NaOH; *k*_non_ = 1.27 × 10^–6^ min^–1^ M^–1^.

b Not determined.

**Figure 3:**
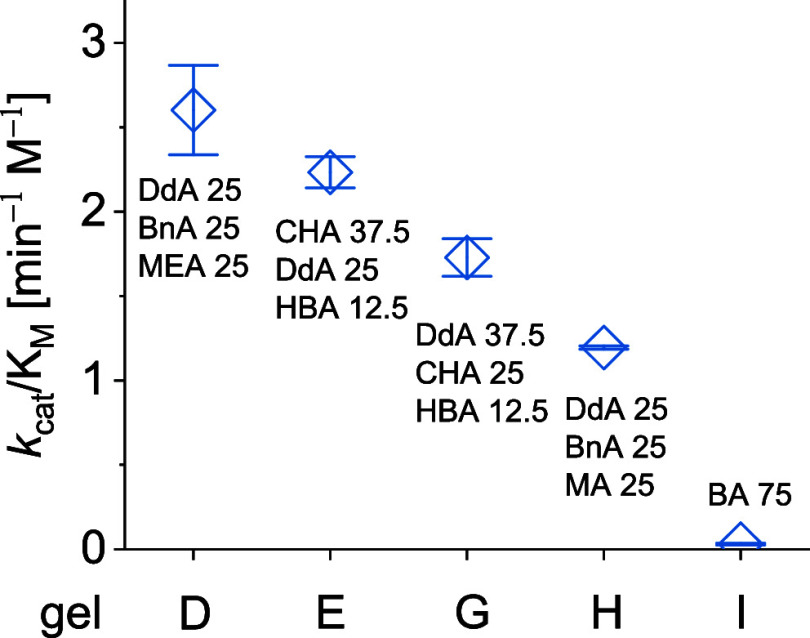
Catalytic efficiency of selected polyacrylate gels with TEGDMA backbone (25 mol %) hydrolyzing maltotriose into maltose and glucose.

The efficiency of the gels for maltotriose cleavage (Table 2) is in the same sequence as toward maltose (Table 1) identifying gel D as the most effective catalyst among all gels studied for the targeted hydrolysis (Figure 3). As observed for maltose hydrolysis, secondary interactions of the material matrix including CH−π and hydrophobic interactions in a TEGDMA-containing gel support the hydrolysis of maltotriose to the largest extent. However, a direct comparison of the catalytic efficiency of the gels across substrates is not meaningful due to the inherently different rates of the uncatalyzed reactions.^30^ In fact, the uncatalyzed hydrolyses of maltotriose and maltose differ by 1 order of magnitude under the given conditions (Tables 1 and 2). Therefore, the catalytic proficiency (*k*_cat_/(*K*_M_ × *k*_non_)) of the catalytic gels is used for a comparison of the gel performances.^31^ Additionally, the stabilization of a transition state can be derived from the reciprocal catalytic proficiency of the reaction.^32^ While gel D has the highest proficiency among the evaluated catalysts, all gels with TEGDMA backbone are about 1.5- to 2-fold more proficient in cleaving a 1→4 α-glycosidic bond in maltotriose than in maltose. The EGDMA-containing gels are likewise about 1.3- to 1.7-fold more proficient for clearing maltotriose over maltose. The stabilization of the transition state upon hydrolysis of maltotriose by gel D reaches 5 × 10^–7^ M and is thus about 2 orders of magnitude over the previously attained stabilization of 4.3 × 10^–5^ using gel I as a catalyst. While gels D and I are synthesized from identical amounts of TEGDMA cross-linker, gel I lacks the synergy of aromatic and strongly hydrophobic interactions that gel D has.

**Figure 4:**
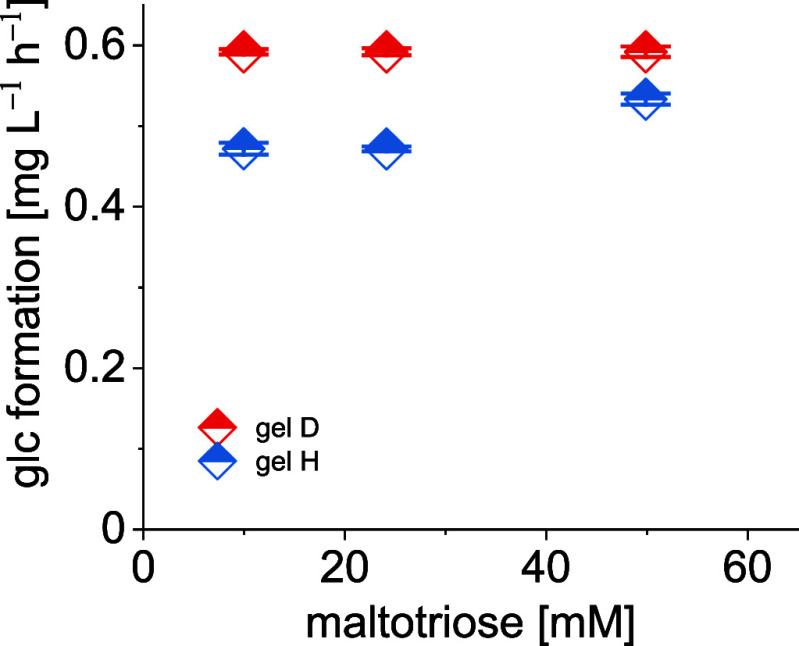
Glucose formation by gel-catalyzed hydrolysis of maltotriose under steady-state conditions.

The formation of glucose by stepwise hydrolysis of maltotriose is complex and involves competing reactions of the maltotriose substrate and the initial maltose product for the catalytic sites of the gels. Thus, kinetic data for the complete hydrolysis of maltotriose have not been obtained. However, the overall glucose amount observed from catalyzed maltotriose hydrolyses using gels D and H is given at selected concentrations as representative examples (Figure 4). The stagnant glucose amount observed for concentrations at steady-state conditions indicates product inhibition, as frequently observed in enzymatic reactions.

## Conclusions

In this proof-of-concept study, a library of 300 gels with unique compositions was synthesized and evaluated for their catalytic proficiency toward the cleavage of 1→4 α-glycosidic bonds. The gels were obtained by UV-initiated radical polymerization of miniemulsions in 12-well plates. The acrylate matrix consists of two series of gels either of EGDMA (**1**, 60 mol %) or TEGDMA (**2**, 25 mol %) cross-linkers and corresponding amounts of acrylate monomer mixtures. While both cross-linkers enable H-bond accepting interactions, the combination of 10 acrylate monomers are systematically altered so that CH−π, hydrophobic, and H-bond donating and accepting secondary interactions are available during catalytic turnover. This approach mimics the features and roles of typical amino acid residues in the active sites of glycosylases to stabilize the transition state of the targeted hydrolysis.

Dynamic light scattering identified 111 monomodal gels in the 300 member library. Out of those, five gels with high potential to cleave the 1→4 α-glycosidic bond in the disaccharide maltose were identified using a spectrophotometric 96-well plate screening assay based on Schiff-base formation of glucose with toluidine. Subsequently, the selected gels and several controls were evaluated in combined hydrolysis and HPLC assays to deduce kinetic parameters. The results allowed the determination of the gel efficiency for the hydrolysis of the targeted glycosidic bond in the disaccharide maltose and the trisaccharide maltotriose. The combined kinetic data furthermore underlined the stability of glycosidic bonds in oligo- and polysaccharides that are increasingly difficult to hydrolyze with a growing number of glycosyl units.

By contrast to previously rationally designed polymers, polyacrylate gel catalysts developed in this approach show their ability to hydrolyze the glycosidic bond in maltotriose with a 1.5-fold higher proficiency than for the same bond in maltose. The difference from previously rationally designed catalysts is found in enabled CH−π stacking and strong hydrophobic interactions by substituting previously used butyl acrylate monomer with mixtures of benzyl and dodecyl acrylate in an empirical approach. The transition state stabilization of the most proficient polyacrylate catalyst in this study (gel D) reaches 5 × 10^–7^ upon hydrolysis of the underivatized oligosaccharide maltotriose equivalent to other approaches with rationally designed catalysts targeting the hydrolysis of activated glycosidic bonds.^10,14^ Overall, the results in this study place the described polyacrylates among the most efficient and proficient man-made catalysts known so far for the hydrolysis of underivatized di- and oligosaccharides. The developed strategy may pave the way toward the development of polyacrylate gels that are able to transform biomass into valuable synthons in the near future.

## Supporting Information Available

Experimental details, materials and methods for gel synthesis, DLS analysis, spectrophotometric assay, UV spectra, and kinetic studies (PDF)
