# Supplementary material for: Developing Catalysts for the Hydrolysis of Glycosidic Bonds in Oligosaccharides Using a Spectrophotometric Screening Assay
Source: ACS Catal. 2024 Aug 14;14(17):12940–6. doi: 10.1021/acscatal.4c03261 (PMC11385356; doi:10.1021/acscatal.4c03261)
Supplement: Supplementary file 1 — cs4c03261_si_001.pdf [file cs4c03261_si_001.pdf]

Developing catalysts for the hydrolysis of glycosidic bonds in oligosaccharides using a spectrophotometric screening assay

Susanne Striegler*

Department of Chemistry and Biochemistry, 345 North Campus Walk, University of Arkansas, Fayetteville, Arkansas, 72701, United States

Email: Susanne.striegler@uark.edu; phone: +1-479-575-5079; fax: +1-479-575-4049

Content **1**

1 Experimental details **2**
1.1 Instrumentation **2**
1.2 Methods, Materials, and Reagents **2**
1.3 Gel synthesis by radical polymerization in 12-well plates **3**
1.3.1 Stock solutions 3
1.3.2 Preparation of pre-polymerization mixtures and polymerization 4
1.4 Gel characterization by dynamic light scattering **4**
1.4.1 Sample preparation 4
1.4.2 Sample characterization 4
1.5 Gel characterization by a spectrophotometric screening assay **5**
1.5.1 Toluidine reagent 5
1.5.2 Gel-catalyzed hydrolysis of di- and oligosaccharides 5
1.5.3 Toluidine assay 5
1.5.4 Representative example of a 96-well plate after the toluidine assay (**Figure S1**) 5
1.5.5 Polymer characterizations by gravimetric analysis for determination of the degree of polymerization and isothermal titration to determine the metal ion reloading 5
1.5.6 UV-Vis spectra of selected carbohydrates after reaction with toluidine reagent (**Figure S2**) 6
1.6 Kinetic hydrolysis assays **7**
1.6.1 Stock solutions 7
1.6.2 Hydrolysis assay 7
1.6.3 HPLC assay 7
1.6.4 Kinetic assay and analysis 7
2 Experimental data **8**
2.1 Monomodal polyacrylate gels from EGDMA crosslinker: composition, dispersity, and hydrodynamic diameters (Table S1) **8**
2.2 Monomodal polyacrylate gels from TEGDMA crosslinker: composition, dispersity, and hydrodynamic diameters (Table S2) **9**
2.3 Figures of the intensity-weighted means of the particles sizes of gels A-I **12**
Intensity-weighted particle size mean for gels A-C synthesized from EGDMA (**1**) (**Figure S3**) 12
Intensity-weighted particle size mean for gels D-I synthesized from TEGDMA (**2**) (**Figure S4**) 13
3 References **14**

1 Experimental details

1.1 Instrumentation

Intensity-weighted hydrodynamic diameters were obtained on a Malvern Panalytical Zetasizer Nano ZS equipped with a 632.8 nm He-Ne laser, a Malvern Zetasizer NanoSampler, a standard Quartz Flow Cell, and the Malvern Zetasizer Software, version 7.13, for data collection and analysis. Spectrophotometric assays were observed in 96-well plate assays using a FilterMax F5 Multi-Mode Microplate Reader from Molecular Devices in absorbance mode equipped with SoftMax Pro 6 software version 6.3. UV–vis spectra were recorded at 25 ± 0.1 ºC over a range of 400–800 nm on a Varian Cary 50 with WinUV Analysis Suite software, version 3.0. Catalytic hydrolyses of maltose and maltotriose were monitored using a HPLC system from Shimadzu equipped with a SCL-10Avp system controller, 2 LC-20AD analytical pumps, DGU-20A3R three channel online degassers, SIL-20A UFLC autosampler with 96 well capability, CTO-20A/ prominence column oven, ELSD-90LT light scattering detector and the LC solution software, version 1.25 for data recording and analysis. A Digital Sonifier (Branson) with flat cap was used for ultrasonication. A medium-pressure lamp TQ 150 (Heraeus Noblelight) with a current of 2.0 A, a voltage of 90 V, and an outer diameter of 13.50 mm was used for free radical polymerizations. Nanopure water at a resistance of 18.2 mΩ was obtained from a ThermoScientific Barnstead E-pure™ water purification system.

1.2 Methods, Materials, and Reagents

For dynamic light scattering, samples were supplied in 2 mL standard clear glass vials for the autosampler supplied with the Malvern Zetasizer instrument; all data were acquired and analyzed using the Malvern Zetasizer Software Version 7.13. Data were recorded using a measuring angle of 173° for back scattering at 20 °C after an equilibration time of 120 s before the first measurement. The data were recorded for settings of the material as a polystyrene latex with a refractive index of 1.590 and nanopure water as the dispersant. Each sample was measured ten times as an average of 10 accumulations each over a 10s duration without delay between measurements. The flow cell is cleaned between measurements three times before acquiring data for additional samples. The reported data are given as an average of ten measurements per sample. For recording absorbance spectra, a semi-micro quartz cuvette (190–900 nm) with a 1 cm thickness, 10 mm path length and 1 mL volume is used. The cell is cleaned between measurements by rinsing with 1,3-propanediol. The spectra are given as an average of data recorded from 400-800 nm over three cycles with a scan rate of 400 nm/min, an average time of 0.1 s, and a 0.67 nm data interval. The sugar content in sample aliquots from kinetic HPLC assays was monitored using a Luna Amino column (Ø 4.6 × 250 mm) from Phenomenex and 80% acetonitrile as the eluent at a flow rate of 1.0 mL/min at 30 °C. Clear 96-well polystyrene microplates (Greiner Bio-One) were used for absorbance assays. Heat-resistant polyester films (VWR) were used as 96-well plate adhesives. Absorbance data for the spectrophotometric screening assay are recorded at 620 nm at 30 °C as an endpoint read. Spectra/Por membranes (Spectrum Labs) with a molecular weight cut-off (MWCO) of 15,000 were used for dialysis. The membranes were soaked in nanopure water for at least 30 min prior to use. pH values were measured using a Beckman Φ 250 pH meter equipped with a refillable ROSS Orion combination pH electrode with a 165 mm long epoxy body, a 95 mm long semi-micro tip and an 8 mm diameter. The pH meter was calibrated before each set of readings. All buffer solutions were prepared by standard methods at ambient temperature accounting for temperature differences at their intended use.

All chemicals are obtained from commercial suppliers and used as received if not noted otherwise. *N*-cyclohexyl-3-aminopropanesulfonic acid (CAPS), 2,2’-dimethoxy-2-phenylacetophenone, copper(II) acetate monohydrate, sodium hydroxide, D-mannose and D-maltose from Sigma-Aldrich; triethylene glycol dimethacrylate (**2**, TEGDMA), tert.-butyl acrylate (**3c**), 2-phenoxyethyl acrylate (**3g**), 2-

methoxyethyl acrylate (**3h**, MEA), 2-hydroxyethyl acrylate (**3i**, HEA), 4-hydroxybutyl acrylate (**3k**, HBA), n-hexadecane, TWEEN 80 and SPAN 80 from TCI America; ethylene glycol dimethacrylate (**1**, EGDMA), n-dodecyl acrylate (**3b**, DdA), methyl acrylate (**3d**, MA), benzyl acrylate (**3f**, BnA) and o-toluidine from Alfa Aesar; 1,3-propanediol, 1,2-dichloroethane and thiourea from Beantown Chemicals (BTC); butyl acrylate (**3a**, BA), neutral aluminum oxide from Acros Organics; acetic acid and sulfamic acid from EMD Millipore; maltotriose and cyclohexyl acrylate (**3e**, ChA) from Thermo Scientific; dimethylsulfoxide from Pharmco-Aarper; and methanol from Supelco. o-Toluidine was distilled in vacuum and stored at -20 °C prior to use. All acrylates are purified by filtration over neutral alumina immediately prior to use. The polymerizable ligand *N,N'*-1,3-bis[(2-hydroxy-4-vinylbenzyloxy)benzylideneamino]propan-2-ol (VBbsdpo, **4**) is synthesized as described,[1] mp = 172-175 °C.

1.3 Gel synthesis by radical polymerization in 12-well plates

1.3.1 Stock solutions

TWEEN/SPAN/CAPS buffer solutions. Initially, 5 mM CAPS buffer solutions are prepared from 0.5571g in 500mL for use at pH of 10.50 at 0 °C by adjusting the pH with aqueous sodium hydroxide solution to pH 10.08 at 23°C following standard procedures.

For polymerization assays using Cu_2VBbsdpo and EGDMA, aqueous TWEEN/SPAN/CAPS emulsions with HLB values of 11 are prepared with an overall weight of 500g from 7.20g of SPAN 80 and 12.2g of TWEEN 80 in 5 mM CAPS buffer as described.[2] For polymerization assays using Cu_2VBbsdpo and TEGDMA crosslinker, aqueous emulsions with an HLB values of 12 are prepared with an overall weight of 200g from 2.53g of SPAN 80 and 6.21g of TWEEN 80 in 5 mM CAPS buffer. The TWEEN/SPAN/CAPS mixtures are stirred at ambient temperature for 30 min and subsequently sonicated at 40 % amplitude (5 sec on, 2 sec off) for 1 min in the cold. All resulting emulsions are kept at ambient temperature and stirred until use. Typically, 2 mL aliquots of the TWEEN/SPAN/CAPS buffer solutions are used during the polymerizations.

Crosslinker/monomer/hydrophobe stock solutions. In a typical procedure, a stock solution of combined 0.3500 mmol of acrylates is prepared consisting of a constant amount of crosslinker (EGDMA: 60 mol%, 0.2100 mmol, 198.2 µL; TEGDMA: 25 mol%, 0.0875 mmol, 114.7 µL) and systematically altered amounts of acrylate monomers.

The monomer mixtures are derived from butyl acrylate, *tert.*-butyl acrylate, benzyl acrylate, cyclohexyl acrylate, dodecyl acrylate, methyl acrylate, 4-hydroxybutyl acrylate, 2-methoxyethyl acrylate, 2-phenoxyethyl acrylate. and 2-hydroxyethyl acrylate. The acrylate monomers are systematically altered to account for 40 mol% for polymerizable acrylate amount synthesized from EGDMA and 75 mol% for polyacrylates synthesized from TEGDMA. The pre-polymerization mixtures are obtained by systematically varying and using all 10 monomers. For polymers containing EGDMA, monomer mixtures of 40/0, 30/10, 20/20, 20/10/10 and 10/10/10/10 mol% are evaluated. For gels containing TEGDMA, monomer mixtures of 75/0, 50/25, 25/25/25 and 37.5/25/12.5 mol% are studied.

For each gel composition, mixtures of 5-times of the needed acrylate amount per well are prepared. Finally, 10 µL of hexadecane are added to the acrylate solutions and mixed. The acrylate/hydrophobe mixtures are prepared immediately prior to polymerization and used in two separate aliquots to synthesize each gel composition in duplicate. The acrylate/hydrophobe pre-polymerization mixtures have typical volumes between 280 and 320 µL, of which aliquots between 55 and 75µL are used per well.

VBbsdpo stock solution. In a typical experiment, VBbsdpo ligand (0.1374 g, 244.4 µmol) was dissolved in DMSO yielding a solution with a total weight of 1.7246 g that is 8 wt% in ligand. The solution was stored at -20 °C until use, thawed, and used in 11.2 µL aliquots per well.

Cu(II) acetate stock solution. In a typical experiment, copper(II) acetate monohydrate (177.01 mg, 886.60 µmol) was dissolved in 5 mL water, stored at ambient temperature, and used in 20 µL aliquots.

Mannose stock solution. In a typical experiment, mannose (633.69 mg, 3.5205 mmol) was dissolved in 10 mL water, stored at ambient temperature, and used in 50 µL aliquots.

Initiator stock solution. Immediately prior to use, 2,2'-dimethoxyphenylacetophenone (0.3935 g, 1.535 mmol) is dissolved in 1620 µL of methanol, stored in the dark at ambient temperature, and used in 40 µL aliquots.

1.3.2 Preparation of pre-polymerization mixtures and polymerization

In all assays, a total of 0.35 mmol polymerizable monomers and 0.5 mol % of *in-situ* formed polymerizable Cu(II) complex are used for synthesizing gels in 2mL TWEEN80/SPAN80 surfactant mixtures in 12-well plates. In a typical procedure, 12-well plates are filled successively with 2 mL of the respective TWEEN/SPAN/CAPS buffer emulsions, an aliquot between 55 and 75 µL of crosslinker/monomer/hydrophobe solution depending on monomer composition, followed by 11.2 µL of ligand stock solution, 20 µL of Cu(II) salt stock solution, 50 µL of mannose stock solution, and 40 µL of initiator solution. The mixtures in each well are then sonicated for 30 s each on ice with a sonication horn at 40 % amplitude (5 sec on, 2 sec off). Subsequently, the plates are subjected to UV light in a 15 cm distance to the source in uncovered plates while stirring the miniemulsions in the wells in the cold over 30 min. The resulting gels are subsequently removed from the 12-well plates and stored in 2 mL vessels at ambient temperature until further use.

1.4 Gel characterization by dynamic light scattering

1.4.1 Sample preparation

In a typical procedure, a 200 µl aliquot of the synthesized gel dispersion is diluted with 800 µl of nanopure water. The diluted solution was initially extracted with 1,2-dichloroethane (2mL, 3x) and then further diluted by addition of 1000 µL of nanopure water. The resulting solution is further sequentially diluted to 1/1250 and used as such for analysis of the particle size and dispersity index of the gel suspension by dynamic light scattering.

1.4.2 Sample characterization

Data acquisition. All samples were supplied in 2 mL standard autosampler vials. Data for the determination of the mean hydrodynamic diameter were obtained at 20 °C using a measuring angle of 173° for back scattering after an equilibration time of 120 s before the first measurement. The data were recorded for settings of the material as a polystyrene latex with a refractive index of 1.590 and nanopure water as the dispersant. Each sample was measured in duplicate as an average of 10 accumulations each over a 10 s duration time without delay between measurements. The flow cell was cleaned with nanopure water between measurements three times before acquiring data for the next sample.

Data analysis. The reported intensity, volume and number mean data are given as an average of the obtained accumulated measurements per sample.

1.5 Gel characterization by a spectrophotometric screening assay

1.5.1 Toluidine reagent

The reagent is prepared by dissolving thiourea (100 mg) in acetic acid (25 mL) at ambient temperature, followed by addition of freshly distilled o-toluidine (5 mL) and sulfamic acid (50 mg). The resulting solution is filled up to 100 mL with 1,3-propanediol and thereby turning yellow. The resulting reagent is stored in a brown glass bottle at -20 °C until to use.

1.5.2 Gel-catalyzed hydrolysis of di- and oligosaccharides

A 50 µL aliquot of the synthesized gels is diluted 1/4 (v/v) with nanopure water. A 25 µL aliquot of the resulting diluted solution is added to 180 µL of nanopure water, followed by 25 µL of a 50 µM solution of the selected carbohydrate stock solution. The resulting solution is stirred magnetically and heated to 60 °C for 30-45 min followed by 20 µL of a 10 mM aqueous sodium hydroxide solution to initiate the reaction. The resulting solutions are stirred for additional 24 h, cooled and stored at -20 °C until use.

1.5.3 Toluidine assay

Each well in the 96-well plate is filled with sugar-gel solution (25 µL) from the gel-catalyzed hydrolysis of di- and oligosaccharides and 100 µL toluidine reagent. The plate is then heated to 120°C for 20min. Subsequently, 75µL of the green solutions are transferred to another plate, the absorbance is read at 620nm. Finally, the plates are scanned to preserve an image of the color distribution as seen by the naked eye.

1.5.4 Representative example of a scanned 96-well plate after the toluidine assay

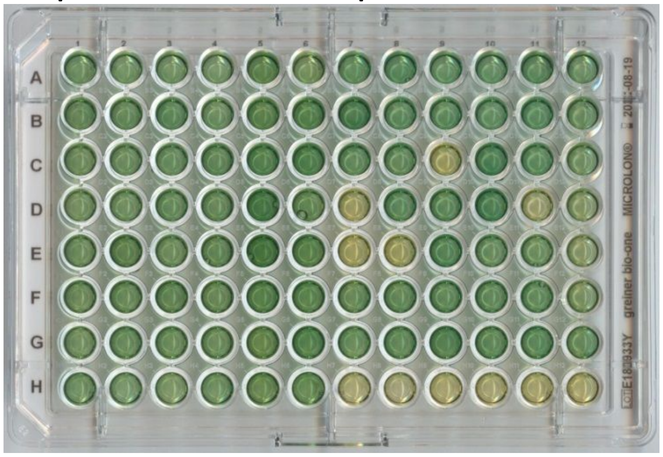

Figure S1. Representative example of a toluidine assay in 96-well plate; each well represents a separate hydrolysis of maltose with a polyacrylate gel as catalyst

1.5.5 Notes on polymer characterizations by gravimetric analysis for determination of the degree of polymerization and isothermal titration to determine the metal ion reloading and catalyst content

Previous extensive efforts to characterize the gel formation in 12-well plates showed by gravimetric analysis near quantitative polymerization for each polymer studied when the polymerization proceeded for 30 min or longer.[3] The elaborated procedure defined the standard polymerization procedure in 12-well plates used here. Therefore, gravimetric analyses with the pre-polymerization mixtures applied are not performed.

Additional previous efforts showed quantitative reloading of gels with immobilized VBbsdpo ligand by Cu(II) ions by isothermal titration calorimetry. A representative study with gel C is published.[2] For all polymers here, quantitative metal ion reloading for all polymers is anticipated based on previous results.

1.5.6 UV-Vis spectra of selected carbohydrates after reaction with toluidine reagent

For measurements of absorbance spectra, 16 wells in two columns of the 96-well plate are filled with 25 µL of 5 mM carbohydrate stock solution followed by 100 µl of toluidine reagent. The mixtures are heated to 120 °C for 20 min and combined. Subsequently, 400 µL of the heated reagent solution are diluted with 600 µL of 1,3-propanediol.

The arbitrary absorbance units of the diluted mixtures are then measured in dependence of the wavelength in three cycles with a scan rate of 400 nm/min and a data interval of 0.67 nm between 400 and 800 nm at 23 °C; the obtained absorbance spectra are given as an average of all measurements. The glucose containing solutions appear green to the naked eye, the solutions containing maltose and reagent only appear yellow.

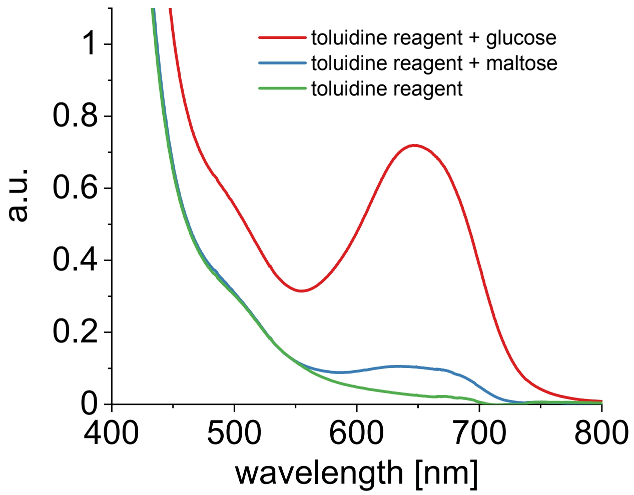

Figure S2. UV spectra of selected carbohydrates after reaction with the toluidine reagent and 1/4 (v/v) dilution of the reaction mixture with 1,3-propanediol

1.6 Kinetic hydrolysis assays

1.6.1 stock solutions

Polyacrylate gel stock solutions. Typically, 1mL aliquots of the synthesized gel dispersions are purified by dialysis and activated by addition of Cu(II) acetate as described.[2-5] The gel stock solutions are then 1.75 mM and are used in 250 µL aliquots for the 2500µL hydrolysis assay.

Carbohydrate stock solutions. In a typical procedure, carbohydrate stock solutions at 25, 50, 75, and 100 mM are prepared in 5 mM volume and used in 250 µL aliquots accounting for final concentrations of 2.5-10 mM in the hydrolysis assay. For higher concentrations, corresponding amounts of carbohydrate are weight in directly into the reaction vessel to account for 15, 20, 25, 30 and 50 mM concentrations in the hydrolysis assay.

1.6.2 Hydrolysis assay

In a typical procedure, 250 µL of a carbohydrate stock solution are added to a dispersion of 250 µL of polyacrylate gel in 1800 µL of nanopure water. For higher sugar concentrations, the weight-in amounts are dissolved in 250 µL of nanopure water to keep the overall volume of the assay constant. Typically, eight separate solutions with nominal carbohydrate concentrations of 2.5, 5.0, 7.5, 10, 20, 25, 30 and 50 mM result. The solutions are equilibrated at 60 °C for 30-45 min before addition of 200 µL of 10 mM sodium hydroxide solution to start the reaction. Subsequently, 100 µL aliquots of the reaction mixtures are taken every 15 min over a total time period of 1.5h, treated with 100 µL of 12.88 mM aqueous hydrochloride acid solution, flash frozen in liquid nitrogen, and stored at -20 °C until analysis of the sugar content.

1.6.3 HPLC assay

The sugar content of each sample aliquot is observed on a Shimadzu system with ESLD detector using a Luna amino column at 30 °C as the stationary phase, and 80 % of isocratic acetonitrile at 1mL/min as the eluent. After thawing the frozen samples and centrifugation at 8500 rpm for 10 min, the supernatant was analyzed for the sugar content in 15 µL aliquots for injection. Under these conditions, maltotriose elutes at 29.2 min, maltose at 15.2 min, and glucose at 7.4 min. The amount of saccharide hydrolyzed, and of monosaccharide formed, was determined from the observed peak areas by comparison to calibration curves from authentic samples.

1.6.4 Kinetic assay and analysis

For analysis of the kinetic performance of the selected polyacrylate gels, initial slopes are determined from the amount of glucose and maltose formed per time and plotted over the respected substrate concentration. The plot of the slopes over the substrate concentration allows determination of the kinetic parameters by the Michaelis-Menten method.

2 Experimental data

2.1 Monomodal polyacrylate gels from EGDMA crosslinker: composition, dispersity, and hydrodynamic diameters

Table S1. Particle size and dispersity of monomodal gels synthesized from EGDMA at selected monomer mixtures

code	Composition [mol %]	PDI	$\overline{X}_{INT} \pm \Delta\overline{X}_{INT}$ [nm]	$\overline{X}_{VOL} \pm \Delta\overline{X}_{VOL}$ [nm]	$\overline{X}_{NUM} \pm \Delta\overline{X}_{NUM}$ [nm]
Monomer mixture [60/40 mol %]					
Gel 1, 812A01	EGDMA/BA (gel C)	0.242	77.9 ± 1.7	68.5 ± 1.1	38.8 ± 3.1
Gel 2, 812A06	EGDMA/MEA	0.129	83.9 ± 0.6	71.0 ± 1.5	54.7 ± 2.6
Gel 3, 812A07	EGDMA/DdA	0.168	82.8 ± 1.0	64.5 ± 0.8	46.6 ± 3.4
Gel 4, 812A09	EGDMA/BnA	0.146	81.9 ± 0.8	68.3 ± 1.1	52.6 ± 1.9
Monomer mixture [60/30/10 mol %]					
Gel 5, 812B07	EGDMA/BA/BnA	0.118	86.9 ± 0.6	75.0 ± 0.6	58.1 ± 2.3
Gel 6, 812B11	EGDMA/BA/HBA	0.117	85.5 ± 0.4	73.7 ± 1.4	56.9 ± 2.4
Gel 7, 812C03	EGDMA/ChA/MEA	0.121	86.2 ± 0.9	74.2 ± 1.4	57.8 ± 1.3
Gel 8, 812C07	EGDMA/ChA/BnA	0.126	86.5 ± 0.6	74.6 ± 1.7	58.7 ± 2.9
Gel 9, 812D03	EGDMA/MEA/ChA	0.133	86.4 ± 0.6	72.8 ± 0.9	55.3 ± 0.4
Gel 10, 812D06	EGDMA/MEA/DdA	0.155	90.2 ± 0.9	75.5 ± 2.6	55.9 ± 3.7
Gel 11, 812D07	EGDMA/MEA/BnA	0.104	86.4 ± 0.1	76.1 ± 0.9	60.4 ± 1.7
Gel 12, 812D09	EGDMA/MEA/MA	0.126	82.3 ± 0.5	69.8 ± 1.1	54.3 ± 2.5
Gel 13, 812D12	EGDMA/MEA/HBA	0.156	79.8 ± 0.8	63.9 ± 0.6	49.2 ± 2.2
Gel 14, 812E06	EGDMA/DdA/MEA	0.100	80.0 ± 0.6	69.2 ± 1.1	54.9 ± 2.1
Gel 15, 812F06	EGDMA/DdA/MEA	0.141	88.1 ± 0.6	75.1 ± 1.2	59.0 ± 2.9
Monomer mixture [60/20/20 mol %]					
Gel 16, 812G06	EGDMA/MA/MEA	0.150	79.8 ± 0.8	66.6 ± 1.2	53.2 ± 3.5
Gel 17, 812G07	EGDMA/MA/DdA	0.149	81.2 ± 0.9	67.8 ± 1.0	52.0 ± 1.5
Gel 18, 812G11	EGDMA/MA/HBA	0.177	65.0 ± 0.8	48.6 ± 3.6	36.0 ± 3.3
Gel 19, 812L06	EGDMA/MEA/DdA	0.133	81.9 ± 0.6	67.7 ± 1.2	51.5 ± 1.7
Gel 20, 812L12	EGDMA/MEA/HBA	0.152	93.5 ± 0.4	78.2 ± 0.7	59.2 ± 2.0
Gel 21, 812N06	EGDMA/MEA/BnA	0.122	92.5 ± 0.5	81.8 ± 1.5	64.0 ± 3.0
Gel 22, 812I06	EGDMA/BA/DdA	0.161	79.7 ± 0.5	64.3 ± 0.1	49.8 ± 4.0
Gel 23, 812K07	EGDMA/BnA/ChA (gel A)	0.111	84.5 ± 0.4	73.1 ± 0.9	57.6 ± 1.7
Monomer mixture [60/10/10/10/10 mol %]					
Gel 24, 812PO6	EGDMA/BA/ChA/MEA/MA (gel B)	0.131	87.0 ± 0.3	74.1 ± 0.5	56.9 ± 1.7

2.2 Monomodal polyacrylate gels from TEGDMA crosslinker: composition, dispersity, and hydrodynamic diameters

Table S2. Particle size and dispersity of monomodal gels synthesized from TEGDMA at selected monomer mixtures

code	Composition [mol %]	PDI	$\overline{X}_{INT} \pm \Delta\overline{X}_{INT}$ [nm]	$\overline{X}_{VOL} \pm \Delta\overline{X}_{VOL}$ [nm]	$\overline{X}_{NUM} \pm \Delta\overline{X}_{NUM}$ [nm]
Gel 25, 802C01	TEGDMA [100 mol%]	0.098	104.0 ± 0.3	94.2 ± 0.5	72.0 ± 1.3
Monomer mixture [25/75 mol %]					
Gel 26, 804A06	TEGDMA/MEA	0.128	86.1 ± 0.6	72.5 ± 2.2	54.4 ± 3.8
Gel 27, 799A03	TEGDMA/PhOEtA	0.106	76.9 ± 0.6	66.2 ± 1.2	52.7 ± 1.7
Gel 28, 802C07	TEGDMA/MA	0.131	70.6 ± 0.4	58.1 ± 0.7	45.1 ± 0.8
Gel 29, 799A01	TEGDMA/BA (gel I)	0.134	71.9 ± 0.5	60.1 ± 0.8	48.0 ± 1.4
Gel 30, 799A07	TEGDMA/^{t}BuA	0.160	70.8 ± 0.6	55.2 ± 0.9	41.3 ± 1.6
Gel 31, 799A05	TEGDMA/ChA	0.095	73.7 ± 0.2	64.4 ± 1.2	52.4 ± 2.1
Gel 32, 799A09	TEGDMA/BnA	0.087	77.2 ± 0.5	67.7 ± 1.2	55.3 ± 2.3
Gel 33, 804A07	TEGDMA/DdA	0.140	93.2 ± 0.9	79.6 ± 1.1	58.2 ± 1.7
Monomer mixture [25/50/25 mol %]					
Gel 34, 799G01	TEGDMA/BA/ChA	0.164	68.4 ± 0.3	52.7 ± 1.4	40.4 ± 2.0
Gel 35, 799G03	TEGDMA/BA/MEA	0.104	71.1 ± 0.4	60.7 ± 1.2	48.3 ± 2.6
Gel 36, 799G05	TEGDMA/BA/DdA	0.168	60.8 ± 0.5	46.5 ± 1.2	35.6 ± 2.0
Gel 37, 799G07	TEGDMA/BA/BnA	0.095	67.3 ± 0.5	57.1 ± 1.8	45.6 ± 2.4
Gel 38, 799G09	TEGDMA/BA/MA	0.129	63.0 ± 0.3	51.7 ± 1.0	40.6 ± 1.9
Gel 39, 799G11	TEGDMA/BA/HBA	0.140	63.9 ± 0.7	51.4 ± 1.2	39.7 ± 1.9
Gel 40, 799H02	TEGDMA/BA/PhOEtA	0.115	69.4 ± 0.4	58.2 ± 0.9	46.1 ± 1.4
Gel 41, 799H03	TEGDMA/BA/^{t}BuA	0.096	69.2 ± 0.3	58.8 ± 1.0	46.8 ± 1.5
Gel 42, 802B01	TEGDMA/BA/HEA	0.182	123.4 ± 1.7	111.4 ± 9.3	66.9 ± 7.6
Gel 43, 799E02	TEGDMA/BnA/BA	0.122	67.1 ± 0.4	55.3 ± 1.0	43.1 ± 1.6
Gel 44, 799E03	TEGDMA/BnA/ChA	0.119	67.2 ± 0.6	55.3 ± 1.4	42.9 ± 1.8
Gel 45, 799E05	TEGDMA/BnA/MEA	0.137	67.0 ± 0.3	55.2 ± 0.8	43.7 ± 1.7
Gel 46, 799E10	TEGDMA/BnA/PhOEtA	0.143	72.0 ± 0.7	58.1 ± 0.4	44.2 ± 0.9
Gel 47, 799E11	TEGDMA/BnA/HBA	0.124	72.3 ± 0.7	59.9 ± 0.9	46.3 ± 1.3
Gel 48, 798F03	TEGDMA/BnA/HEA	0.197	60.8 ± 1.4	83.1 ± 6.2	36.9 ± 1.5
Gel 49, 798F07	TEGDMA/BnA/Dda	0.161	64.3 ± 0.3	50.2 ± 1.4	38.1 ± 1.4
Gel 50, 798F10	TEGDMA/BnA/MA	0.118	57.9 ± 0.3	47.8 ± 0.6	38.5 ± 1.0

code	Composition [mol %]	PDI	$\overline{X}_{INT} \pm \Delta\overline{X}_{INT}$ [nm]	$\overline{X}_{VOL} \pm \Delta\overline{X}_{VOL}$ [nm]	$\overline{X}_{NUM} \pm \Delta\overline{X}_{NUM}$ [nm]
Monomer mixture [25/50/25 mol %]					
Gel 51, 798D10	TEGDMA/MEA/MA	0.152	87.7 ± 0.6	69.4 ± 5.5	46.1 ± 12.1
Gel 52, 799B02	TEGDMA/MEA/BA	0.142	68.8 ± 0.3	56.1 ± 1.0	43.5 ± 1.4
Gel 53, 799B03	TEGDMA/MEA/PhOEtA	0.102	69.0 ± 0.5	57.8 ± 1.6	44.8 ± 1.5
Gel 54, 799B06	TEGDMA/MEA/DdA	0.182	69.0 ± 0.9	50.8 ± 0.9	35.6 ± 1.0
Gel 55, 799B07	TEGDMA/MEA/BnA	0.106	71.3 ± 0.4	60.2 ± 1.4	47.5 ± 2.5
Gel 56, 799B09	TEGDMA/MEA/ChA	0.094	74.0 ± 0.3	63.5 ± 0.5	50.6 ± 0.7
Gel 57, 799B11	TEGDMA/MEA/HBA	0.186	74.7 ± 1.0	56.4 ± 3.9	39.6 ± 3.8
Gel 58, 802B03	TEGDMA/MEA/HEA	0.189	86.0 ± 0.5	67.8 ± 4.1	49.1 ± 3.7
Gel 59, 798H02	TEGDMA/HBA/BA	0.096	62.8 ± 0.3	53.2 ± 0.7	42.9 ± 1.1
Gel 60, 798H06	TEGDMA/HBA/MEA	0.130	65.0 ± 0.1	52.9 ± 0.8	41.0 ± 1.4
Gel 61, 798H09	TEGDMA/HBA/BnA	0.139	60.1 ± 0.5	48.5 ± 1.2	39.2 ± 1.5
Gel 62, 798H12	TEGDMA/HBA/MA	0.170	124.7 ± 0.7	117.9 ± 3.4	71.4 ± 5.0
Gel 63, 802B11	TEGDMA/HBA/HEA	0.204	129.2 ± 2.3	122.8 ± 2.5	68.0 ± 4.8
Gel 64, 799H06	TEGDMA/HBA/ChA	0.144	66.4 ± 0.4	51.9 ± 0.8	38.5 ± 0.7
Gel 65, 799H08	TEGDMA/HBA/PhOEtA	0.147	64.3 ± 0.5	50.5 ± 1.5	38.1 ± 2.3
Gel 66, 804B08	TEGDMA/HBA/DdA	0.200	105.4 ± 0.6	84.4 ± 0.6	42.4 ± 0.6
Gel 67, 799C01	TEGDMA/PhOEtA/BA	0.120	73.2 ± 0.4	60.2 ± 0.9	45.9 ± 1.2
Gel 68, 799C03	TEGDMA/PhOEtA/MEA	0.123	75.0 ± 0.8	62.7 ± 0.8	48.8 ± 1.0
Gel 69, 799C05	TEGDMA/PhOEtA/DdA	0.104	79.3 ± 0.8	68.3 ± 1.4	53.8 ± 2.2
Gel 70, 799C07	TEGDMA/PhOEtA/BnA	0.121	72.8 ± 0.7	61.0 ± 1.0	47.7 ± 1.5
Gel 71, 799C09	TEGDMA/PhOEtA/MA	0.139	72.7 ± 0.5	59.4 ± 2.0	45.6 ± 3.4
Gel 72, 799C12	TEGDMA/PhOEtA/HBA	0.147	67.4 ± 0.3	54.2 ± 0.7	41.9 ± 1.1
Gel 73, 799E07	TEGDMA/DdA/ChA	0.106	86.8 ± 0.7	74.6 ± 1.7	57.3 ± 3.1
Gel 74, 799F01	TEGDMA/DdA/BA	0.169	66.5 ± 0.3	51.9 ± 4.8	36.0 ± 2.1
Gel 75, 799F03	TEGDMA/DdA/PhOEtA	0.126	71.7 ± 0.2	58.4 ± 1.7	44.2 ± 2.4
Gel 76, 799F06	TEGDMA/DdA/MEA	0.151	69.4 ± 0.4	54.9 ± 0.5	41.2 ± 0.7
Gel 77, 799F08	TEGDMA/DdA/BnA	0.170	69.9 ± 0.4	52.7 ± 0.4	37.9 ± 1.0
Gel 78, 799F10	TEGDMA/DdA/MA	0.137	74.0 ± 0.4	68.4 ± 2.0	42.9 ± 2.5

code	Composition [mol %]	PDI	$\overline{X}_{INT} \pm \Delta\overline{X}_{INT}$ [nm]	$\overline{X}_{VOL} \pm \Delta\overline{X}_{VOL}$ [nm]	$\overline{X}_{NUM} \pm \Delta\overline{X}_{NUM}$ [nm]
Monomer mixture [25/50/25 mol %]					
Gel 79, 799D01	TEGDMA/ChA/BA	0.146	71.0 ± 0.3	57.8 ± 1.1	45.1 ± 1.6
Gel 80, 799D03	TEGDMA/ChA/MEA	0.096	73.8 ± 0.4	63.6 ± 1.4	51.0 ± 2.1
Gel 81, 799D05	TEGDMA/ChA/DdA	0.110	84.8 ± 0.5	72.5 ± 0.7	55.0 ± 0.6
Gel 82, 799D07	TEGDMA/ChA/BnA	0.106	75.2 ± 0.3	64.1 ± 1.0	50.6 ± 1.3
Gel 83, 799D09	TEGDMA/ChA/MA	0.106	72.2 ± 0.7	61.1 ± 1.1	48.9 ± 1.7
Gel 84, 799D11	TEGDMA/ChA/HBA	0.127	71.0 ±0.4	58.8 ± 0.4	45.5 ± 0.6
Gel 85, 802C08	TEGDMA/MA/MEA	0.114	71.7 ± 0.3	60.2 ± 0.4	47.1 ± 1.0
Gel 86, 802C11	TEGDMA/MA/HBA	0.105	54.3 ± 0.5	54.3 ± 0.8	42.8 ± 1.1
Gel 87, 798G02	TEGDMA/MA/BA	0.181	126.9 ± 1.1	119.6 ± 4.2	70.3 ± 8.2
Gel 88, 798G08	TEGDMA/MA/DdA	0.285	144.7 ± 1.7	87.4 ± 5.4	26.2 ± 7.7
Gel 89, 798G09	TEGDMA/MA/BnA	0.173	81.1 ± 0.5	62.1 ± 2.4	43.4 ± 3.4
Gel 90, 799H10	TEGDMA/MA/ChA	0.152	67.1 ± 0.3	52.3 ± 0.6	38.8 ± 1.1
Monomer mixture [25/25/25/25 mol %]					
Gel 91, 802E02	TEGDMA/HBA/MA/BA	0.134	75.6 ± 0.4	62.7 ± 0.9	48.3 ± 2.2
Gel 92, 802E04	TEGDMA/HBA/MA/HEA	0.135	84.1 ± 1.0	70.1 ± 1.8	52.0 ± 3.0
Gel 93, 802E05	TEGDMA/HBA/MA/MEA	0.121	74.8 ± 0.4	62.3 ± 2.2	48.1 ± 3.3
Gel 94, 802E08	TEGDMA/HBA/MA/DdA	0.117	77.8 ± 0.3	66.2 ± 1.5	51.8 ± 2.3
Gel 95, 802E10	TEGDMA/HBA/MA/BnA	0.155	71.6 ± 0.5	56.9 ± 1.3	43.2 ± 1.4
Gel 96, 806F02	TEGDMA/HBA/BA/ChA	0.187	74.5 ± 0.3	55.0 ± 0.8	41.3 ± 2.0
Gel 97, 804C04	TEGDMA/HBA/BA/MEA	0.215	86.9 ± 0.9	64.1 ± 1.9	42.1 ± 1.5
Gel 98, 804C07	TEGDMA/HBA/BA/DdA	0.226	88.1 ± 0.4	62.4 ± 3.7	40.6 ± 0.8
Gel 99, 804C10	TEGDMA/HBA/BA/BnA	0.127	91.2 ± 0.7	79.2 ± 1.0	61.6 ± 2.8
Gel 100, 804D03	TEGDMA/HBA/MEA/ChA	0.208	84.8 ± 0.9	62.0 ± 1.9	46.4 ± 2.1
Gel 101, 804D06	TEGDMA/HBA/MEA/DdA	0.126	93.6 ± 0.2	81.6 ± 1.1	62.8 ± 2.3
Gel 102, 804D08	TEGDMA/HBA/MEA/BnA	0.191	76.6 ± 0.6	53.2 ± 0.8	44.4 ± 2.5
Gel 103, 804E07	TEGDMA/HBA/ChA/DdA	0.167	88.1 ± 0.8	70.1 ± 0.4	47.5 ± 1.3
Gel 104, 804F08	TEGDMA/HBA/ChA/BnA	0.301	79.1 ± 0.8	39.5 ± 4.7	22.8 ± 3.2

code	Composition [mol %]	PDI	$\overline{X}_{INT} \pm \Delta\overline{X}_{INT}$ [nm]	$\overline{X}_{VOL} \pm \Delta\overline{X}_{VOL}$ [nm]	$\overline{X}_{NUM} \pm \Delta\overline{X}_{NUM}$ [nm]
Monomer mixture [25/25/25/25 mol %]					
Gel 105, 923C03	TEGDMA/DdA/BnA/MEA (gel D)	0.216	110.8 ± 0.4	82.5 ± 0.8	34.3 ± 0.8
Gel 106, 923D01	TEGDMA/DdA/BnA/MA (gel H)	0.254	100.4 ± 0.2	60.6 ± 2.1	27.4 ± 0.5
Monomer mixture [25/37.5/25/12.5 mol %]					
Gel 107, 806B01	TEGDMA/ChA/BA/HBA	0.194	65.9 ± 0.6	47.8 ± 0.8	35.6 ± 1.9
Gel 108, 806B03	TEGDMA/ChA/DdA/HBA (gel E)	0.191	72.7 ± 0.8	53.3 ± 0.8	37.5 ± 1.3
Gel 109, 923D09	TEGDMA/DdA/BnA/HBA (gel F)	0.246	103.0 ± 0.1	93.8 ± 1.7	52.4 ± 1.1
Gel 110, 806C04	TEGDMA/DdA/ChA/HBA (gel G)	0.155	76.0 ± 0.8	60.3 ± 0.1	44.7 ± 0.1
Gel 111, 806E07	TEGDMA/MA/BnA/HBA	0.257	67.0 ± 0.1	47.2 ± 1.3	33.3 ± 6.5

2.3 Figures of the intensity-weighted means of the particles sizes of gels A-I

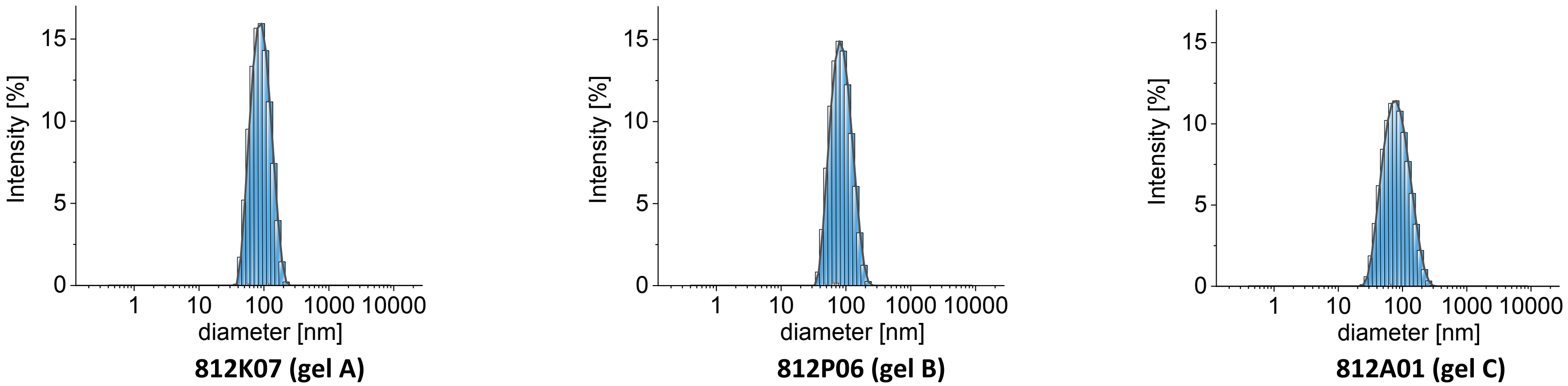

Figure S3. Intensity-weighted particle size mean for gels A-C synthesized from EGDMA (**1**)

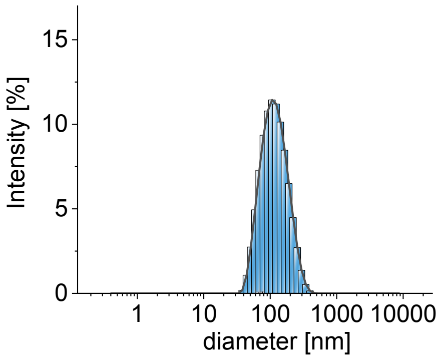

923C03 (gel D)

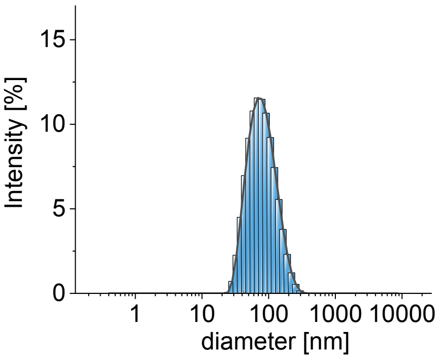

806B03 (gel E)

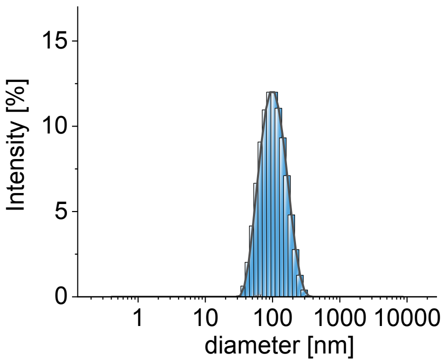

923D09 (gel F)

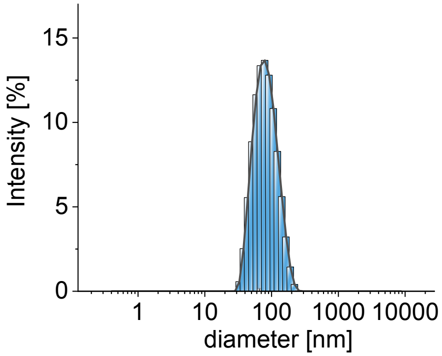

806D04 (gel G)

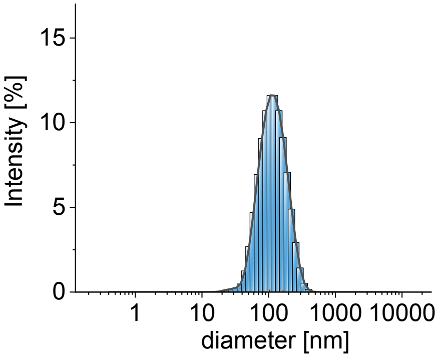

923D01 (gel H)

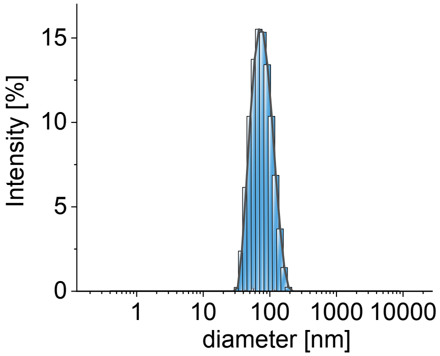

799A01 (gel I)

Figure S4. Intensity-weighted particle size mean for gels D-I synthesized from TEGDMA (**2**)

3 References

1. Striegler, S.; Gichinga, M. G.; Dittel, M., Macromolecular Salen Catalyst with Largely Enhanced Catalytic Activity *Org. Lett.* **2008,** 10 (2), 241-244.

2. Sharma, B.; Striegler, S., Nonionic Surfactant Blends to Control the Size of Microgels and Their Catalytic Performance during Glycoside Hydrolyses *ACS Catal.* **2020,** 10 (16), 9458-9463.

3. Sharma, B.; Striegler, S., Nanogel Catalysts for the Hydrolysis of Underivatized Disaccharides Identified by a Fast Screening Assay *ACS Catal.* **2023,** 13 (3), 1614-1620.

4. Sharma, B.; Striegler, S., Polarity and critical micelle concentration of surfactants support the catalytic efficiency of nanogels during glycoside hydrolyses *ACS Catal.* **2022,** 12 (15), 8841–8847.

5. Striegler, S.; Sharma, B.; Orizu, I., Microgel-catalyzed hydrolysis of non-activated disaccharides *ACS Catal.* **2020,** 10 (24), 14451-14456.
